# Universal Programmable Portable Measurement Device for Diagnostics and Monitoring of Industrial Fluid Power Systems

**DOI:** 10.3390/s21103440

**Published:** 2021-05-15

**Authors:** Ryszard Dindorf, Piotr Wos

**Affiliations:** Department of Mechanical Engineering and Metrology, Kielce University of Technology, 25-314 Kielce, Poland; wos@tu.kielce.pl

**Keywords:** portable measuring device, diagnostic measurement, fluid power systems, leakage flow

## Abstract

This paper presents a new universal programmable portable measuring device (PMD) as a complete, accurate, and efficient solution for monitoring and technical diagnostics of industrial fluid power systems. PMD has programmable functions designed for recording, processing, and graphical visualization of measurement results at the test stand or the place of operation of fluid power systems. PMD has a built-in WiFi communication module for transferring measurement data via Industrial Internet of Things (IIoT) technology for online remote monitoring of fluid power systems. PMD can be programmed for a variety of measuring tasks in servicing, repairing, diagnosing, and monitoring fluid power systems. For this purpose, the fluid dynamic quantity, mechanical quantity, and electrical quantity can be measured. The adjustment of the PMD to the indirect measurement of leakage flow rate in a compressed air system (CAS) is presented in detail. Measuring instruments and PMDs were connected to a branch of the pipeline. The tests used the measurement system to estimate the leakage flow rate through air small nozzles, as well as other CAS indicators.

## 1. Introduction

In condition monitoring and technical diagnostics of industrial fluid power systems (hydraulic and pneumatic), measurements using various sensors, transducers, and measurement sets are carried out. In [1], the steps of monitoring fluid systems have been defined. Diagnostic measurements are needed to compare the actual—instantaneous—state with the reference state to determine the suitability or unfitness of a technical object, as well as to determine the cause of the existing state and to make a prediction of the future states of the object. The purpose of diagnostic measurements of fluid power systems is to determine their technical condition and quality of operation. Condition monitoring (CM) of fluid power systems to minimize machine downtime and optimize maintenance work is increasingly used in industry, mobile machinery, power engineering, among others. CM of fluid power systems in the field may be difficult due to various environmental conditions (temperature fluctuations, varying levels of contaminates), improper operation, and variable payload cycles. CM of complex production systems, from a single hydraulic or pneumatic machine to the entire production and assembly line, is possible thanks to the use of intelligent sensors and measurement systems [2]. Measuring instruments used in the diagnostics of fluid power systems are used to measure such parameters as pressure, flow rate, temperature, contamination and state of fluids, power consumption, vibrations, and acoustic emission [3]. For the implementation of various diagnostic tasks, measurement sets are used, which include an electronic measuring device, computer software, measuring transducers, and measuring equipment. Electrical signals from the measuring sensors are processed using the measurement protocol, displayed on charts, printed, or sent to a computer. It is also possible to set the physical units, measuring range, average, maximum, and minimum reading values, as well as to define different relationships between the measurement data, e.g., from two sensors of the same type (pressure drop). Various measurement modes are also possible: normal measuring, fast curve recording, and long-term measuring. Available measurement kits for the diagnostics of fluid power systems have a limited number of measurement channels; they enable simultaneous measurement of only basic parameters such as pressure, pressure difference, temperature, and flow rate. Portable measuring devices can be used for measuring tasks in various fluid technologies, such as hydraulics, pneumatics, lubrication process, refrigeration, and air conditioning. A portable measuring device allows basic measurements to be performed, the processing of measurement data, and the implementation of measurement procedures. The HMG 4000 Portable Data Recorder, in addition to recording the current parameter values, enables the average, maximum, and minimum values to be read, as well as the different relationships calculated from the readings of the two transducers [4]. MultiHandy 3020 facilitates diagnostics of pressure systems, and its software offers a wide range of measurement functions [5]. DS 500 mobile is an intelligent chart recorder designed to monitor compressed air systems (CAS) [6]. Measurement data, parameter values, curves, threshold exceedances, and alarms are recorded for remote reading by a web server. Based on this, various indicators useful in the CAS audit can be calculated. Service Master Plus (SMP) is a diagnostic test kit for measuring, recording, and analyzing data of any hydraulic or pneumatic system [7]. The main disadvantage of commercial measuring devices used in the diagnosis and monitoring of fluid power systems is the inaccessibility of the user interface for programming or configuring these devices. The user interface should enable the choosing of inputs, data processing mode, recording options, and additional functions, such as alerting states, displaying various information, communication with other measuring devices, as well as wireless access to the network, password protection, etc.

Portable measuring devices are increasingly used in science, engineering, research, and industry. The latest generation of portable measuring devices combines the advantages of a mobile application with the reliability and precision of a stationary measuring device. They are multi-functional systems with an interface for computer-aided data acquisition and recording. Portable measurement devices are usually not the subject of study, and there are no reports of individual designs for such devices. There are also few reports on the use of portable measurement devices in scientific studies. A practical method of calibration of a robot based on an innovative wireless portable measuring device was presented in [8]. Article [9] proposes a portable measuring device based on a UVI sensor, with which the general public can have easy access to information related to UV radiation. There is more medical research using portable measuring instruments. The paper [10] presents an objective portable measuring device with two indenter probes for spinal joint accessory motion testing (JAMT).

Electronic portable instruments are built for specific applications that have standard measuring functions with user interface, signal processing, and communication capabilities [11]. Portable devices can communicate wirelessly with other digital devices and computers over communication networks. The addition of smart sensors brings the benefits of machine-to-machine communication that supports the Industrial Internet of Things (IIoT) or smart factories (Industry 4.0).

## 2. Universal Programmable PMD

**Figure 1:**
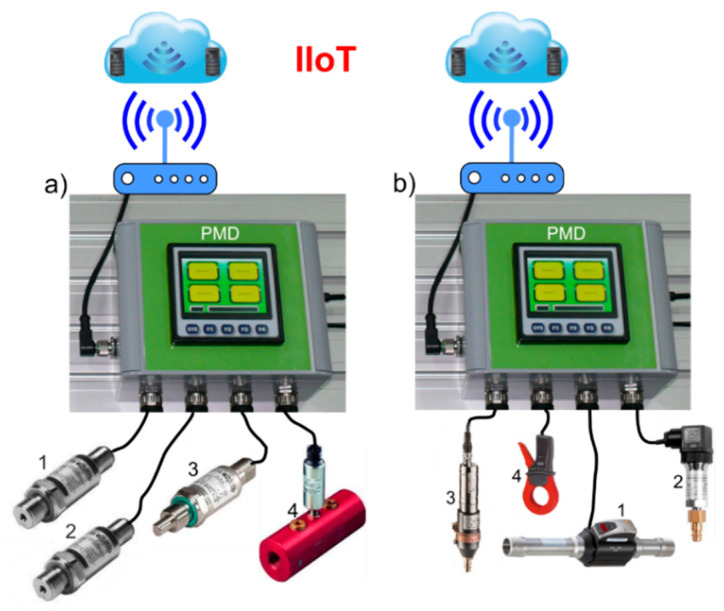
Two PMD measurement sets: (**a**) for diagnostics of hydraulic systems: 1—pressure sensor, 2—pressure sensor, 3—temperature sensor, 4—flow meter, (**b**) for monitoring of compressed air systems: 1—flow counter, 2—pressure sensor, 3—temperature sensor, dew point sensor, 4—clamp-on ammeters.

A new universal programmable PMD was developed and tested. Figure 1 shows two PMD measurement sets used for diagnostics of hydraulic systems and monitoring of compressed air systems (CAS).

PMD can be adapted to the diagnostic measurements of fluid power systems, in which the most frequently measured are dynamic fluid quantities (pressure, flow rate, temperature), mechanical quantity (position, linear speed, rotational speed, force, torque, shaft power) and electrical quantity (voltage, current, power). PMD relies on fast microcontrollers and microprocessors to process digital signals, which, with small dimensions and low energy consumption, satisfy the computing needs. PMD has a programmable main screen, which includes graphics and digital display bars, menu, function buttons, a clear indication of the operating status, and alarm indicators. The software used is for menu programming, quick device configuration, real-time display of measured values, parameter data processing, and the performance of various calculation functions. Both the device software and the graphics interface have been implemented on the device driver. The recording of measurement data, calculation processes, and editions of spreadsheets are performed in the internal memory. Graphical and text recordings of measured values (charts, tables) can be simultaneously recorded from several measuring channels. Measurement channels and connections can be adapted to various electrical input standards. It is possible to program alarm outputs and operating states related to light, sound, and visual signaling as well as notifications via the Internet. PMD can access external devices with Virtual Network Computing (VNC) software on Windows, Linux, and Mac OS X operating systems. External devices such as desktops and portable computers (laptops, tablets), and smartphones communicate with the PMD via the WiFi wireless access point. Reading the measurement values, setting threshold values (limits) for the process, downloading alarm status reports, and switching alarms on and off are performed by using a Web browser. Through the website, PMD can be remotely configured or data can be transferred from PMD.

**Figure 2:**
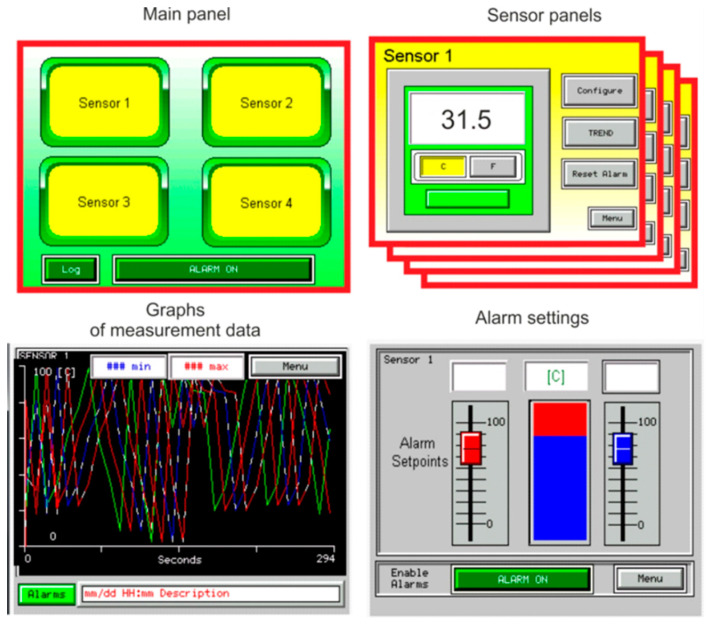
The view of the programmed PMD touch screen.

The view of the programmed PMD touch screen with the main panel, sensor panel, graphics of measurement data, and alarm settings is shown in Figure 2. Four sensors are programmed on the main panel, which can be: HP pressure transducer (600 bar), HT temperature transducer (−50–200 °C), TFM turbine flow meter (300 L/min), or GFM gear flow meter (30 L/min), and RSS rotation speed sensor (10,000 rpm, 166.6 Hz). The implemented calculation functions enable, e.g., the calculation of the efficiency (total, volumetric, hydro-mechanical) of pumps, actuators, and motors, flow parameters, or pressure losses in hydraulic and pneumatic transmission pipelines.

**Figure 3:**
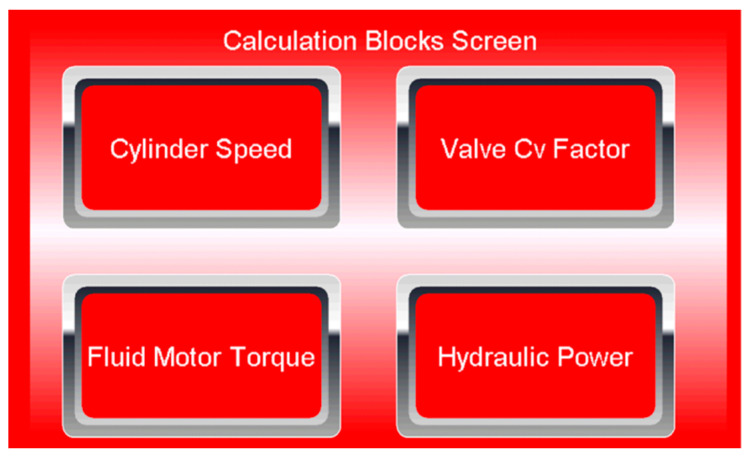
PMD calculation blocks screen.

Figure 3 shows the PMD touch screen with four calculation blocks based on the measurement of flow rate (*q*_v_), pressure (*p*), and rotation speed (*n*). The calculation blocks cylinder speed, valve Cv factor, fluid motor torque, and hydraulic power include the calculation functions *F* = *f*(*q*_v_, *p*, *n*).

The calculation results are displayed directly on the graphics panel, saved in the internal memory, and made available in the network through the built-in WebServer module. As a result, people from anywhere can use the calculation results and monitor the condition of hydraulic power systems.

**Figure 4:**
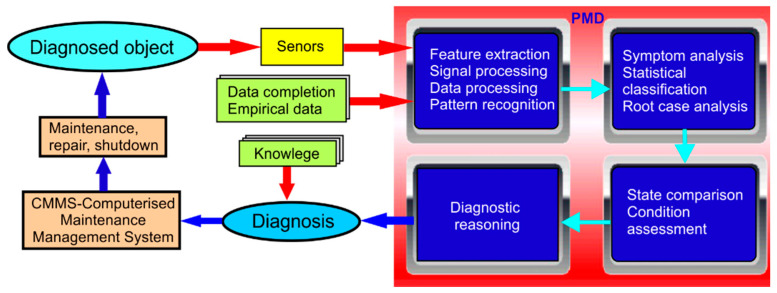
General diagram of the diagnostic process with the use of PMD.

The scientific aspects of PMD are related to the possibility of implementing computational algorithms, automatic measurement algorithms, identification algorithms, and control algorithms for monitoring and diagnostic fluid power systems. It is possible to create specialized applications due to the specific needs of the user. A general diagram of the diagnostic process of the diagnosed object (hydraulic power system) with the use of PMD is shown in Figure 4.

## 3. Adaptation of the PMD to Measure the Leakage Flow Rate in CAS

**Figure 5:**
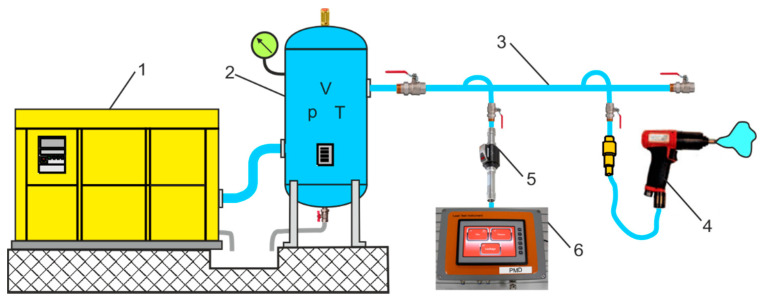
Schematic diagram of CAS: 1—compressor, 2—stand-alone receiver tank, 3—pipeline system, 4—pneumatic tool, 5—measuring instrument, 6—PMD.

Monitoring and diagnostic measurements are of particular importance in compressed air systems (CAS), which are used to analyze the efficient use of compressed air energy (CAE) [12,13]. Assessment of the effective use of CAE should include an analysis of the efficiency of the production and use of compressed air; an assessment of the possibility of reducing compressed air consumption; and the adoption of a CAS improvement cost reduction plan. It is especially important to reduce air consumption and leakage losses, which not only waste energy but also cause a pressure drop in the CAS. An indirect standard method is used to assess leakage in CAS pipelines, which consists of measuring the static pressure drop at any given time interval [14,15]. This measurement method requires the compressor and all unused equipment to be turned off. In previous tests, the authors proposed an indirect method of leakage measurement for a control flow on a branch of the pipeline [16]. This is a noninvasive leak measurement method in CAS that does not require any rework or modification of the pipeline. Measuring instruments are connected parallel to the pipeline, which makes the measurement convenient anywhere in the CAS. The tests used measuring instruments and PMD connected to a branch of the CAS pipeline to estimate the leakage flow rate through the air nozzle of pneumatic tools (Figure 5).

The theoretical fundamental of the indirect method for leakage measurement in CAS pipelines is the principle of mass conservation in the air tank. The mass flow equation in the air tank with constant volume *V* (*V* = const) is as follows

(1) $$q_{m}=\frac{dm}{dt}=\frac{d\left(V/\vartheta \right)}{dt}=\frac{1}{\vartheta \;}\frac{dV}{dt}-\frac{V}{\vartheta^{2}}\frac{d\vartheta }{\;dt}\Rightarrow q_{m}=-\frac{V\;}{\vartheta^{2}}\;\frac{d\vartheta }{dt}$$

where *ϑ* is the specific volume.

From the equation of the polytropic process (exponent *n* in the range 1 ≤ *n* ≤ 1.4), we obtain

(2) $$n\;\frac{d\vartheta }{\vartheta }+\;\frac{dp}{p}=0\Rightarrow \frac{d\vartheta }{dt}=-\frac{\vartheta }{n\;p}\;\frac{dp}{dt}$$

where *p* is the instantaneous air pressure inside the air tank.

From (1) and (2), the mass flow rate *q_m_* and the volumetric flow rate *q*_v_ result

(3) $$q_{m}=\frac{V}{n\;p\;\vartheta \;}\;\frac{dp}{dt}\Rightarrow \;q_{v}=\vartheta \;q_{m\;}=\frac{V}{n\;p\;}\;\frac{dp}{dt}$$

**Figure 6:**
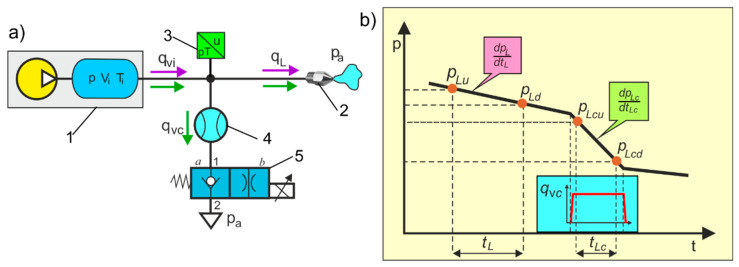
The principle of indirect measurement of leakage flow rate in air piping, (**a**) basic diagram of the measuring system: 1—compressed air station, 2—air nozzle, 3—dual *p*/T transducer, 4—flow meter, 5—2/2-way throttle control valve, normally closed (NC), electrically operated (position *a* valve is closed, position *b* valve is open), (**b**) measurement points of pressure drop ratios and controlled flow rate in two-time intervals.

The principle of indirect measurement of the leakage flow rate *q_L_* through the air nozzles, based on the measurement of the controlled flow rate at the branch of the air piping and the instantaneous pressure rate in two time intervals, is shown in Figure 6.

In the first measurement, without a controlled flow in the branch pipeline, the throttle control valve is closed. The pressure drop ratio *p_Lu_*/*p_Ld_* at the time interval *t_L_* is measured. The compressed air flow equation is as follows

(4) $$\frac{V}{n\;p_{L}}\;\frac{dp_{L}}{dt_{L}}=q_{L}\Rightarrow q_{L}=\;\frac{V}{n\;t_{L}}\ln\left(\frac{p_{Lu}}{p_{Ld}}\right)$$

In the second measurement with a controlled flow in the branch pipeline, the throttle control valve is open. The pressure drop ratio *p_Lcu_*/*p_Lcd_* and the controlled flow rate *q*_v*c*_ at the same time interval *t_Lc_* are measured.

The compressed air flow equation is as follows:

(5) $$\frac{V}{n\;p_{Lc}}\;\frac{dp_{Lc}}{dt_{Lc}}=q_{L}+q_{vc}\Rightarrow q_{L}+q_{vc}=\frac{V}{n\;t_{Lc}}\;\ln\left(\frac{p_{Lcu}}{p_{Lcd}}\right)$$

From (4) and (5), the leakage flow rates *q_L_* in CAS are calculated

(6) $$q_{L}=\;q_{vc}\;\frac{\ln\left(\frac{p_{Lu}}{p_{Ld}}\right)\;t_{Lc}}{\ln\left(\frac{p_{Lcu}}{p_{Lcd}}\right)\;t_{L}-\;\ln\left(\frac{p_{Lu}}{p_{Ld}}\right)\;t_{Lc}}$$

PMD records the measurement results and performs calculations according to the procedure shown in the flow chart [17]. According to this procedure, the corrected leakage flow rate *q_Lm_* is

(7) $$q_{Lm}=\;K_{C}\;K_{T}\;q_{L}$$

where *K_C_* is the calibration factor dependent on the measurement conditions and *K_T_* is the correction factor dependent on the temperature measurement.

(8) $$\;K_{T}=\;\frac{T_{m}}{T_{N}}$$

where *T_m_* is the average measurement temperature.

**Figure 7:**
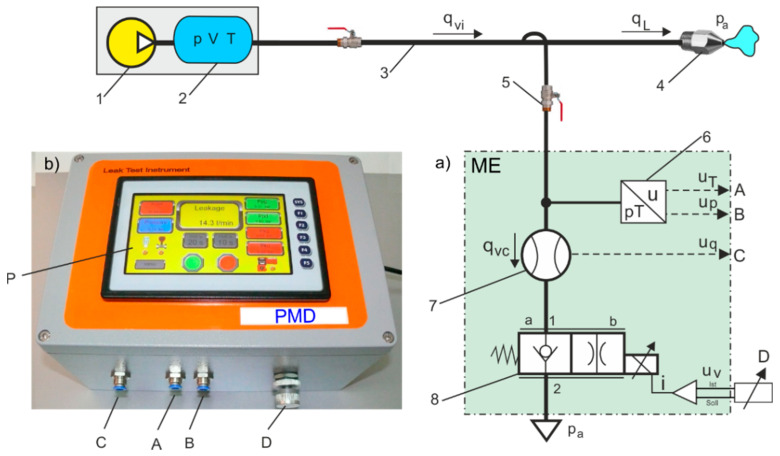
Schematic diagram of a measuring system for indirect leakage measurement on a branch of the pipeline: 1—compressor station, 2—air receiver tank, 3—air pipeline, 4—symbolic of the leak point, 5—shut-off ball valves, (**a**) ME—measurement equipment: 6—dual *p*/*T* transducer, 7—flow meter, 8—proportional throttle valve, (**b**) PMD: P—touch screen, A—connector to a pressure transducer, B—connector to temperature transducer, C—connector to flow meter, D—valve setpoint potentiometer.

As can be seen from (6), the proposed indirect measurement method does not take into account the volume *V* of the air tank, which is not the case with traditional indirect measurement methods. The indirect measurement methods on a branch of the pipeline have been used in laboratory tests to estimate the leakage flow rate in CAS [18]. The authors are the inventors of automated measuring systems (AMS) for leakage flow measurement in gas transmission pipelines [19]. The implementation of the PMD demo version for leakage flow measurements in industrial CASs was co-financed by local EU structural funds. The schematic diagram of an indirect measurement system for the measurement of flow leakage rate through the air nozzle using measurement equipment (ME) and PMD is shown in Figure 7.

The ME comprises a pressure and temperature transducer, a thermal flow meter, and a pneumatic 2/2 (2-way, 2-position) proportional throttle valve. PMD, when connected to ME, reads out the voltage signals *u_T_* and *u_p_* from the dual *p*/*T* transducers (A and B connectors) and the voltage signal *u_q_* from the thermal mass flow meter (C connector). The proportional throttle valve is manually adjusted by the setpoint potentiometer D. Potentiometer D is used to set the flow rate *q*_v*c*_ through the throttle valve. During the measurement of leakage without controlled flow, the throttle valve is closed; PMD records the upper *p_Lu_* and lower *p_Ld_* pressure values in *t_L_*. This allows the pressure drop ratio *p_Lu_*/*p_Ld_* due to leakage *q_L_* in the pneumatic pipeline to be determined. When measuring leakage with the controlled flow *q*_v*c*_, the throttle valve is open, and the PMD records the upper *p_Lcu_* and lower *p_Lcd_* pressure values in *t_Lc_*. This allows the pressure drop ratio *p_Lcu_*/*p_Lcd_* due to leakage *q_L_* in the pneumatic pipeline to be determined. All measuring sensors cooperating with PMD have analog current outputs in the range of 4–20 mA and 0–10 V for specific measuring ranges. Based on each of the measurement points, it is possible to create a programmable calibration curve with the possibility of introducing corrections during the calibration of the measuring channel. Tuning (adjustment) during the use of a programmable PMD is possible using a software change in the value of the calibration curve coefficients. It is possible to make corrections (tuning) from the touch panel of the device. In comparison with the standard multifunctional measuring instruments available on the market, the proposed programmable PMD enables the measuring device to be set with the smallest possible deviation from the correct value. The measurement uncertainty of an instrument depends on all apparatus errors, but mainly on the measuring sensors used in measurements. The mass flow rate *q_mc_* is measured with a TSI 4043 thermal flow meter (*q*_v*n*_ = 200 L/min = 0.00033 m^3^/s); pressure *p_m_* and temperature *T_m_* are measured with a dual ATM/T transducer (*p_n_* = 10 bar = 1 MPa, *T_n_* = 373.15 K). The accuracy of the thermal mass flowmeter is *eq_m_* = ±2% of readout, the accuracy of the pressure transducer is *ep_m_* = ±0.5% FS, and the temperature transducer is *eT_m_* = ±1%/1.5 K. The accuracy of the calibration of the measuring system is performed for the range of leakage flow rate in CAS pipeline and the range of flow rate through the throttle control valve.

The measuring system uses a throttle control valve, the flow rate of which should be adjusted to the leakage flow through the measuring nozzle. When selecting a throttle control valve from the catalog, its two parameters are used, namely the acoustic conductivity, *C* sonic conductance, and *b* critical pressure ratio. These two parameters describe the flow rate properties in detail and are proposed by the currently applicable ISO standard [20]. The measurement must be carried out in the range of the choked flow in the control valve. It follows that the pressure drop time *t_c_* in the choked flow of the control valve must be longer than the controlled flow time *t_Lc_*, *t_c_* > *t_Lc_*.

For adiabatic air discharge through the control valve, the flow equation is as follows

(9) $$q_{mc}=-\frac{V}{\kappa \;R\;T_{m}}\;\frac{dp}{dt}$$

where *κ* is the adiabatic exponent, *R* is the individual gas constant for air, *T_m_* is the average measurement temperature, *p* is the measurement pressure, and *q_mc_* is the mass flow rate through the throttle control valve

(10) $$q_{mc}(p)=C\;\rho_{N}\;p\;\sqrt{T_{N}/T_{m}}$$

In the range of choked flow in the valve, the pressure drops from the initial pressure *p_in_* to the critical pressure *p_c_* = (1 − *b*) *p_in_* at the time from *t_c_*. After integrating (9), we get

(11) $$\int_{0}^{t_{c}}dt=-\frac{V}{\kappa \;R\;T_{m}}\;\int_{p_{in}}^{p_{c}}\frac{dp}{q_{mc}(p)}\;=-\tau \;\int_{p_{in}}^{p_{c}}\frac{dp}{p}$$

where *τ* is the time constant $\tau =\frac{V}{\kappa \;R\;T_{m}\;C\;\rho_{N}\;\sqrt{T_{N}/\;T_{m}}}$, *ρ_N_* and *T_N_* are the density and temperature under standard conditions.

The solution of the integral Equation (11) gives the formula to calculate the pressure drop time in the choked flow of the throttle control valve

(12) $$t_{c}=\tau \;\ln\left(\frac{p_{in}}{p_{c}}\right)=\tau \;\ln\left(\frac{1}{1-b}\right)\;>\;t_{Lc}$$

## 4. Measurement Results

**Figure 8:**
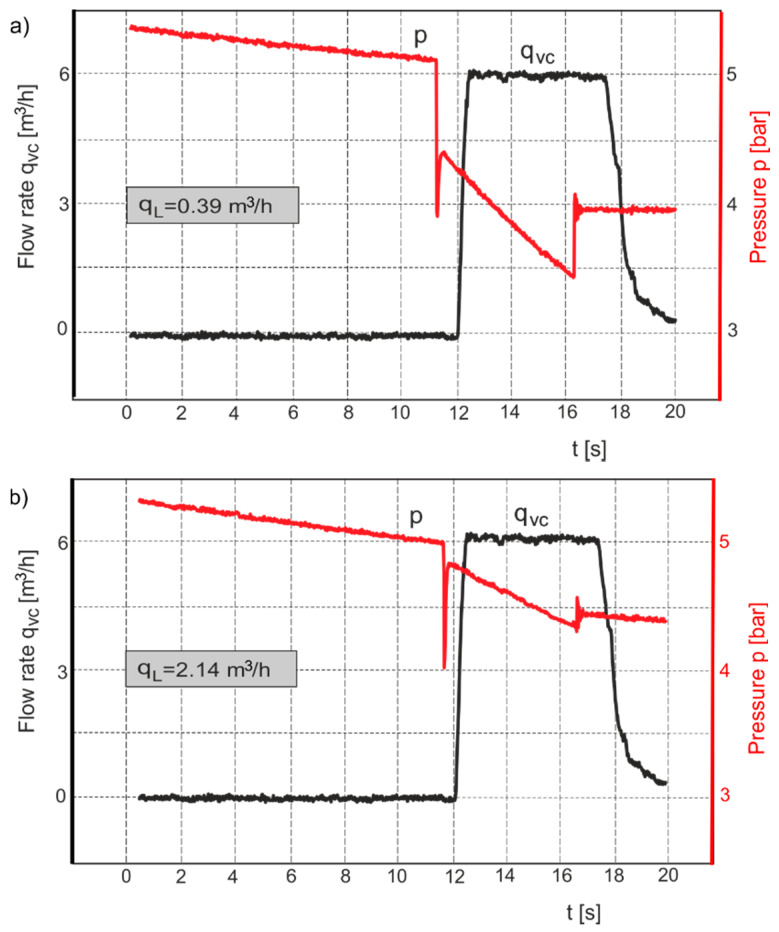
Graphical representation of measurement results for pressure *p*(*t*) and controlled flow rate *q*_v*c*_(*t*) for the selected diameter of the air nozzle, (**a**) *d* = 0.3 mm, (**b**) *d* = 0.7 mm.

The measurement system, with PMD connected to a branch of the pneumatic pipeline, was tested. The flow rate through small air nozzles for the selected two diameters was measured. Figure 8 shows the measurement charts of pressure *p*(*t*) and the controlled flow rate *q*_v*c*_(*t*) for the two diameters of the air nozzles, while Table 1 presents the limit values of the parameters read from the charts and the calculated leakage flow rate value *q_L_*.

Based on the guide and uncertainty in measurement (GUM) [21], the type B standard uncertainty of the controlled flow rate *q*_v*c*_ is calculated as follows

(13) $$\begin{array}{l} u_{B}(q_{vc})=\sqrt{{\left(\frac{{\partial q}_{vm}}{\partial C}\right)}^{2}\;u^{2}(C)+{\left(\frac{{\partial q}_{vm}}{{\partial p}_{m}}\right)}^{2}\;u^{2}(p_{m})+{\left(\frac{{\partial q}_{vm}}{{\partial T}_{m}}\right)}^{2}\;u^{2}(T_{m})}\\ =\sqrt{{\left(p_{m}\;\sqrt{\frac{T_{N}}{T_{m}}}\right)}^{2}\;{\left(\frac{\Delta C}{2}\right)}^{2}+{\left(C\;\sqrt{\frac{T_{N}}{T_{m}}}\right)}^{2}\;{\left(\frac{\Delta p_{m}}{\sqrt{3}}\right)}^{2}+{\left(-\frac{C\;p_{m}\;\sqrt{T_{N}}}{2\;\sqrt{{T_{m}}^{3}}}\right)}^{2}\;{\left(\frac{\Delta T_{m}}{\sqrt{3}}\right)}^{2}}=0.15\;m^{3}/h \end{array}$$

where *u^2^*(*p_m_*) and *u^2^*(*T_m_*) are the variance values of the pressure and temperature measurements; *u^2^*(*C*) is the variance of the sonic conductance estimated with the assumption of the normal probability distribution. According to [22], the uncertainty of sound conductivity *C* is calculated as follows

(14) $$\frac{sC}{C}=\sqrt{{\left(\frac{{sq}_{vm}}{q_{vn}}\right)}^{2}+{\left(\frac{{sp}_{m}}{p_{n}}\right)}^{2}+0.25\;{\left(\frac{{sT}_{m}}{T_{n}}\right)}^{2}}=0.0268$$

where *sq*_v*m*_, *sp_m_*, and *sT_m_* are the experimental standard deviation values of flow rate, pressure, and temperature, respectively. *q*_v*n*_, *p_n_*, and *T_n_* are the nominal values of flow rate, pressure, and temperature, respectively. 

The standard deviation values of *sq*_v*m*_ = 0.03 m^3^/h, *sp_m_* = 0.1 bar, and *sT_m_* = 1.15 K for 40 experiments were calculated. The relative standard uncertainty of the controlled flow rate was determined

(15) $${\delta q}_{vc}=\frac{u_{B}(q_{vc})}{q_{vc}}\;100\%=2.5\%$$

**Figure 9:**
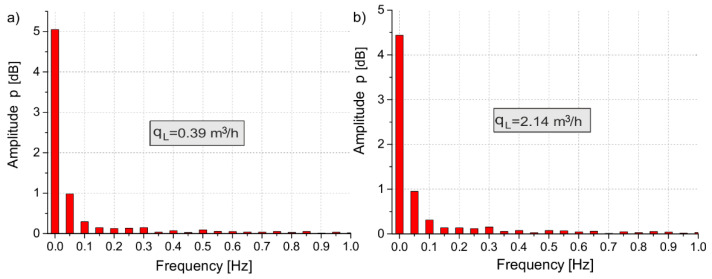
Spectral characteristics of the pressure signals: (**a**) *d* = 0.3 mm, (**b**) *d* = 0.7 mm.

During analyzing the charts of the pressure signals recorded by the measuring system in Figure 8, one can observe disturbances caused by the opening and closing of the 2/2-way throttle control valve. The Discrete Fourier Transform (DFT) algorithm to process the pressure disturbance from the experimental data was used. DFT makes it possible to analyze, study, and synthesize signals in a way that cannot be used in the processing of continuous analog signals [23]. Based on the pressure charts in Figure 8, the spectral characteristics of the pressure signals were determined, as shown in Figure 9.

Frequency analysis of the pressure ratio measurement results shows that the essential disturbance of the measuring signal resulting from the opening and closing of the throttle control valve is within the frequency range of 0.05–0.6 Hz.

**Figure 10:**
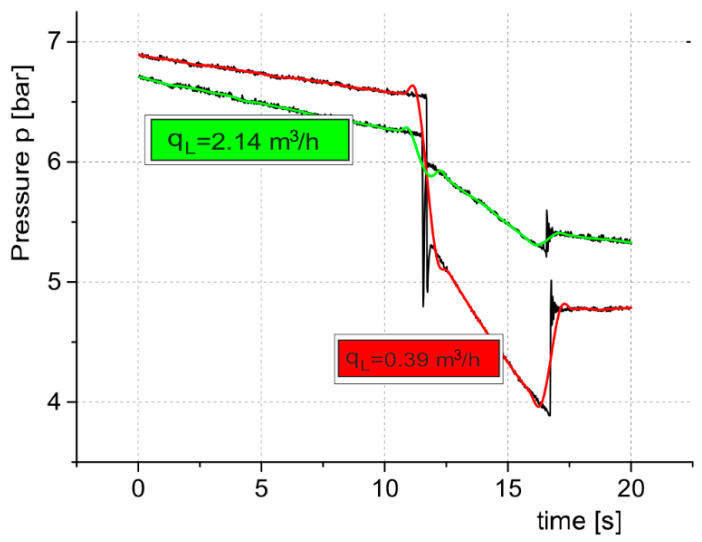
Graphical representation of the pressure *p*(*t*) measurement results with and without digital filtering.

The digital output signals from the pressure transducer are noisy, which makes it difficult to accurately measure especially small pressure changes. In the proposed indirect method, two instantaneous pressure values are measured after switching the control valve. The pressure measurement noise generated is then digitally filtered using the LOESS (Locally Estimated Scatterplot Smoothing) linear regression method [24]. The method used is based on a “classical” method such as linear least squares regression. It is a method that facilitates quick processing of measurement data; therefore, it was effectively used in the developed PMD solution. Figure 10 shows the graphical representation of the pressure *p*(*t*) measurement results with and without digital filtering.

PMD enables the programming of various computational functions for estimating basic CAS indicators, such as leakage loss, pressure drop, energy cost, air consumption cost, etc. [25]. For the measured leakage flow rate through small air nozzles, the annual energy cost (*AEC*) in EUR/yr was estimated as

(16) $$AEC=q_{L}\;SEC\;h_{L}\;ER$$

where *q_L_* is the leakage flow rate in m^3^/h, *SEC* is the specific energy consumption for CAS (acceptable *SEC* = 0.12 kWh/Nm^3^), *h_L_* is the annual leakage flow in hours (full-hour operating system *h_L_* = 8000 h/yr), and *ER* is the energy rate (in Polish industry *ER* = 0.1 EUR/kWh).

The results are as follows: for one air nozzle with diameter *d* =0.3 mm (*q_L_* = 0.39 m^3^/h), the *AEC* = 37.44 EUR/yr, and for one air nozzle with diameter *d* =0.7 mm (*q_L_* = 2.14 m^3^/h), the *AEC* = 205.44 EUR/yr.

## 5. Discussion

**Figure 11:**
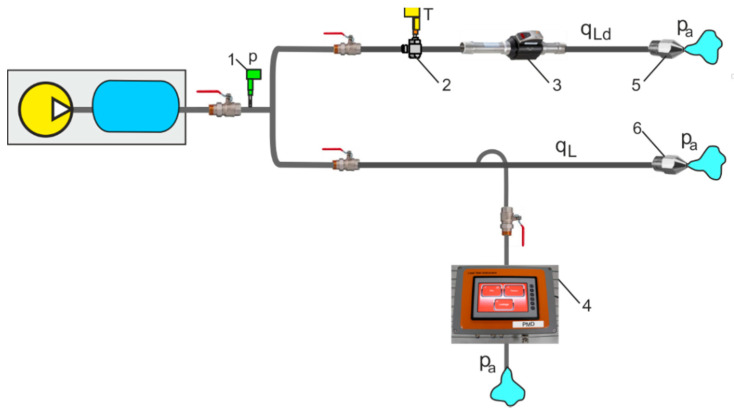
Schematic diagram of direct and indirect leakage flow measurement through blow nozzles: 1—pressure sensor, 2—temperature sensor, 3—thermal mass flow meter, Sensyflow-D, 4—PMD, 5, 6—air nozzles.

To verify the accuracy of the leakage flow measurement in the air nozzle with the indirect method using PMD, direct measurements with the thermal mass flow meter Sensyflow-D, FTM200-D type, manufactured by ABB, were carried out. In the direct leakage flow measurement, the flow meter is installed in the distribution pipeline. The schematic diagram of direct and indirect leakage flow measurement through air nozzles is shown in Figure 11. To compare the direct and indirect measurement results of leakage flow rate through the tested air nozzles, a relative error *δ_L_* according to the equation was calculated

(17) $$\delta_{L}=\left|\frac{q_{L}-q_{Ld}}{q_{Ld}}\right|\;100\%$$

where *q_Ld_* is the leakage flow rate in the direct measurement, and *q_L_* is the leakage flow rate in the indirect measurement.

Table 2 compares the results of measuring the leakage through the air nozzles with the indirect measurement using PMD and the direct measurement with the Sensyflow-D thermal mass flow meter.

By comparing the results of direct and indirect measurements of the leakage flow rate for the tested nozzles, the relative error *δ_L_* was calculated. Measurements were made for the same initial conditions. The relative error did not exceed 5%, which is acceptable in the pneumatic pipeline audit. Measurements were made for the smallest air nozzle diameters, which indicates that the measuring system used (measuring instruments and PMD) is sensitive to small leaks. With a small pressure drop in the nozzle, the temperature of the compressed air in the pipeline remains constant.

## 6. Conclusions

Universal programmable PMDs, as a complete, accurate, and efficient solution for monitoring and technical diagnostics of industrial fluid power systems, were developed and tested. The PMD has the following advantages: flexibility by combining different measurements, versatility through individual solutions for measuring tasks, ease of use thanks to one software package, and compliance with the standards of measuring devices in fluid power systems. It is possible to create specialized applications due to the specific needs of the user. Portable measurement devices are usually not the subject of study, and there are no reports of individual designs for such devices. The disadvantages of PMD are the programming skills of measurement and calculation functions and the need to adapt the input channels to standard sensors from different manufacturers. The extensive programmable functions of PMD allow for individualized identification of the technical condition of each fluid power equipment. PMD provides efficient processing of recorded measurement values, local data storage, data transmission, and processing via wired networks or wireless technology. PMD has a built-in module for wireless communication, which is a key requirement for Industry 4.0 and Industrial Internet of Things (IIoT) technology. PMD has been adjusted for indirect measurement of the leakage flow in the pneumatic pipeline. The programmability, functionality, and accuracy of the PMD were tested when measuring the leakage flow rate through small air nozzles. The usefulness of PMD for the measurement of pneumatic pipeline parameters and their graphical presentation on characteristic charts has been demonstrated. Based on the measurement data, the PMD enables the quantitative assessment of leakage flow rate and financial cost caused by air leaks. The suitability of PMD for monitoring and diagnostic measurements of pneumatic systems was tested in laboratory and industrial conditions by the industrial partner OBR Pneumatyka (Poland). PMD will be used in a research project, which seeks to implement a mobile laboratory for auditing industrial CASs.

## 7. Patents

Patent Number P.426255 2019 (Poland). Device for measuring leakage rates in pipelines for gas transmission, especially compressed air. Inventors Dindorf, R. and Wos, P.

## Figures and Tables

**Table 1:** The results of measurements and calculations from the charts in Figure 6.

*d* mm	*T_m_* K	*p_L_* bar	*t_L_* s	*p_Lc_* bar	*t_Lc_* s	*q*_v*c*_ m^3^/h	*q_L_* m^3^/h
0.3	314	6.8	8	4.7	2	6	0.39
		6.6		4.2			
0.7	314	6.7	8	5.7	2	6	2.14
		6.3		5.3			

**Table 2:** The results of leakage flow measurements through the air nozzle with direct and indirect methods.

*d* mm	*p* bar	*T* K	*q_L_* m^3^/h	*q_Ld_* m^3^/h	*δ_L_* %
0.6	6.6	321	0.324	0.335	3.28
0.8	6.6	321	1.275	1.318	3.26
1.0	6.6	321	1.911	1.976	3.29

## Data Availability

The data presented in this study is available from the respective author upon request. The data is not publicly available due to the non-existence of a publicly accessible repository.
